# Calpain Inhibition Reduces Axolemmal Leakage in Traumatic Axonal Injury

**DOI:** 10.3390/molecules14125115

**Published:** 2009-12-09

**Authors:** Endre Czeiter, András Büki, Péter Bukovics, Orsolya Farkas, József Pál, Erzsébet Kövesdi, Tamás Dóczi, János Sándor

**Affiliations:** 1 Department of Neurosurgery, University of Pécs, Pécs, Hungary; E-Mail: endre.czeiter@aok.pte.hu; 2 Department of Public Health, Institute of Applied Health Sciences University of Pécs, Pécs, Hungary; E-Mail: janos.sandor@etk.pte.hu

**Keywords:** calpain, MDL-28170, traumatic axonal injury, traumatic brain injury

## Abstract

Calcium-induced, calpain-mediated proteolysis (CMSP) has recently been implicated to the pathogenesis of diffuse (traumatic) axonal injury (TAI). Some studies suggested that subaxolemmal CMSP may contribute to axolemmal permeability (AP) alterations observed in TAI. Seeking direct evidence for this premise we investigated whether subaxolemmal CMSP may contribute to axolemmal permeability alterations (APA) and pre-injury calpain-inhibition could reduce AP in a rat model of TAI. Horseradish peroxidase (HRP, a tracer that accumulates in axons with APA) was administered one hour prior to injury into the lateral ventricle; 30 min preinjury a single tail vein bolus injection of 30 mg/kg MDL-28170 (a calpain inhibitor) or its vehicle was applied in Wistar rats exposed to impact acceleration brain injury. Histological detection of traumatically injured axonal segments accumulating HRP and statistical analysis revealed that pre-injury administration of the calpain inhibitor MDL-28170 significantly reduced the average length of HRP-labeled axonal segments. The axono-protective effect of pre-injury calpain inhibition recently demonstrated with classical immunohistochemical markers of TAI was further corroborated in this experiment; significant reduction of the length of labeled axons in the drug-treated rats implicate CMSP in the progression of altered AP in TAI.

## 1. Introduction

Traumatic brain injury (TBI) generates widespread traumatic axonal injury (TAI) in animals and man significantly contributing to mortality and morbidity [1]. In the last two decades, detailed investigation of the pathobiology of TAI has revealed that the majority of injured axons are not mechanically severed at the time of impact as it had been proposed previously, but instead these axons show progressive changes gradually evolving towards axonal disconnection [2,3,4]. These changes include focal axolemmal permeability alterations (APA), induction of calpain-mediated proteolysis (CMSP) and neurofilament sidearm modification leading to neurofilament compaction (NFC) long associated with impaired axoplasmatic transport and axonal swelling that concludes in axonal disconnection [4]. Recent studies demonstrated that the exact pathobiology of TAI should be far more complex than it was assumed previously. Specifically, considerable proportion of injured axons displaying APA and NFC do not swell, and TAI is characterized with different morphological traits and immunohistochemical markers in different fiber tracts [5,6].

Detailed subcellular mechanisms responsible for initiating these progressive changes have also been unmasked over the last several years implicating the involvement of calcium (Ca^2+)^ and calpains through several indirect approaches. Calpains represent a well-conserved family of Ca^2+^-dependent cysteine proteases (for review, see [7]). They consist of several ubiquitous and tissue specific isoforms with broad substrate specificity influencing many aspects of cell physiology including migration, proliferation and apoptosis. It is well documented that calpain activation contributes to various neurodegenerative conditions [8]. Hong *et al.* [9] demonstrated the neuroprotective effect of a cell-penetrating calpain inhibitor administrated systematically. Their findings suggest that targeting of intracellular Ca^2+^-activated mechanisms, such as proteolysis, represents a viable therapeutic strategy for limiting neurological damage. Results of Saatman *et al.* [10] suggest that calpain may play an important role in the pathogenesis of TBI including the degradation of cytoskeletal proteins such as spectrin. According to Banik *et al.* [11] calpain- and free-radical inhibitors may rescue cells and preserve and maintain membrane structure by preventing protein breakdown, this way also preserving motor function. There are further studies that underline this axonoprotective feature of calpain inhibitors, for example in the opossum optic nerve [12] and in the rat spinal cord [13]. Posmantur *et al.* [14] found morphological protection including preservation of dendritic structure and reduction of axonal retraction balls throughout the ipsilateral cortical region in a rat model of TBI applying calpain inhibitor 2.

Recent observations demonstrated axolemmal permeability alterations in foci of TAI that were directly correlated with cytoskeletal and mitochondrial damage, a finding entirely consistent with local Ca^2+^ overload occurring as a result of altered axolemmal permeability [15,16]. When first observed, it was assumed that this APA and presumed Ca^2+^ influx would cause catastrophic and ultrarapid degradation of the intraaxonal cytoskeleton. However, ultrastructural analysis of damaged axonal profiles displaying Ca^2+^-induced, calpain-mediated spectrin proteolysis (CMSP) demonstrated relatively well preserved ultrastructural details for several hours postinjury as well as an unexpected compartmentalization in the accumulation of the immunoreactive end products. Specifically, through the immunohistochemical localization of spectrin breakdown products (SBDP) we have recognized [17], in the early posttraumatic period, that the calpain- mediated structural proteolysis was confound to the subaxolemmal domain, progressing over time to involve the entire extent of the axon-cylinder. On the basis of these observations we posited that the initial mechanoporation of the axolemma should lead to an influx of Ca^2+^, first activating calpain at the subaxolemmal domain where digestion of the spectrin component of the cortical cytoskeleton (subaxolemmal network or membrane skeleton) should lead to further destabilization of the axolemma, resulting in further influx of Ca^2+^ and the activation of an excessive proteolytic cascade that digests the axonal cytoskeleton and other targets of the Ca^2+^ activated cysteine protease, calpain.

CMSP and the accumulation of SBDPs have been demonstrated in traumatically injured humans, too and now these substances are considered potential biomarkers in the care of the head injured [18,19].

In a recently published paper we have reported the application of a cell permeable peptidyl-aldehyde calpain inhibitor (MDL-28170) in an attempt to determine whether this drug attenuates traumatically induced axonal damage to provide further evidence of the role of CMSP in the pathogenesis of TAI, while setting the stage for additional therapeutic studies on the utility of calpain- inhibitors for the treatment of TAI. Our results demonstrated that calpain inhibition led to a significant decrease in the density of damaged axonal profiles displaying altered axoplasmatic transport (APP-IR) and NFC (RMO-14-IR) [20].

The present study was initiated to provide further evidence for a direct association between CMSP and APA via preinjury administration of the calpain inhibitor MDL-28170 that has been combined with preinjury administration of horseradish peroxidase (HRP). We hypothesized that HRP enters the axons that have sustained axolemmal permeability-alteration at the moment of injury (“axolemmal mechanoporation”); then calpain-inhibition should halt further alterations in axolemmal permeability, a phenomenon reflected in decreased length of damaged axonal profiles labeled with HRP in drug treated versus vehicle treated animals.

## 2. Materials and Methods

### 2.1. The Rat Model of Impact Acceleration TBI

Adult Wistar rats (380–400 g, four drug, four vehicle treated) were used for experiments, three animals served as sham-injured controls. After induction of anesthesia rats were intubated and ventilated with a mixture of 1–2% isoflurane in 30% oxygen and 70% dinitrogen-oxide. The skull between the coronal and lambdoid sutures was exposed with a midline incision. A metallic disc-shaped helmet was glued to this point and the animal was placed in a prone position on a foam bed with the helmet centered under the edge of a Plexiglas tube. Brass weights of 450 g were allowed to fall from a height of 200 cm through the tube directly to the disc. Two animals were excluded because of skull fracture. Immediately after the injury the animal was ventilated with 100% O_2_. The helmet was removed and the skull was investigated for any sign of fracture which would have disqualified the animal from further evaluation. The scalp wound was sutured with the animal remaining on artificial ventilation until the predetermined survival of 120 min after injury [21].

### 2.2. Drug Administration

Drug treatment was carried out in a blinded fashion. Horseradish peroxidase (HRP, normally excluded from the cytoplasm by the intact axolemma) was administered one hour prior to injury into the lateral ventricle (IVC administration) and a single tail vein bolus injection of 30 mg/kg MDL-28170 dissolved in 1 ml of the vehicle (PEG300/EtOH, 9:1) was administered 30 min prior to injury (n = 4). The dosage and administration was selected according to recent pharmacokinetic studies [22]. Other rats received bolus injection of the vehicle alone (n = 4). After drug administration gelfoam and bone wax were used to restore skull integrity before the induction of closed head injury. We have not noticed any toxic effect of HRP or the drug administered.

### 2.3. Physiological Parameters

Core- (Physitemp) and temporalis muscle- temperature were continuously monitored (FHC, Bowdoinham, ME, USA) and adjusted to physiological values with a heating pad (FHC) and a lamp. Arterial oxygen saturation was monitored via pulse oxymetry (Nonin 8600V, Nonin Medical, Minneapolis, MN, USA) via the footpad and/or the ear.

### 2.4. Histochemistry

At the predetermined posttraumatic survival time of two hours, animals were euthanized and transcardially perfused with aldehydes (2% paraformaldehyde, 2.5% glutaraldehyde in 0.1 M Millonig's buffer). The brainstem was blocked in the sagittal plane and 50-μm sagittal sections were cut on a vibratome and processed for the visualization of the peroxidase reaction product using the cobalt glucose oxidase method [3]. In this protocol, sections were incubated at 37 °C with 0.05% diaminobenzidine, 0.2% D-glucose, 0.04% ammonium chloride, and glucose oxidase type II (0.41 mg/100 mL) in a 0.1 M Millonig's buffer for two 1-h periods. The sections were mounted on glass slides, dried, dehydrated, and cleared in alcohol and xylene baths, then coverslipped for light microscopic (LM) investigation [23].

### 2.5. Image Analysis

Tissue analysis was performed by an investigator blinded to the nature of the treatment. Six sections were sampled from each animal and one field from each section. All the sections were sampled from predetermined anatomical locations in the pontomedullary junction. HRP-labeled axonal profiles were examined with a NIKON light microscope interfaced with a computer-assisted image analysis system (IMAGE-PRO PLUS 5.0.1) in the area of the corticospinal tracts (CSpT) and the medial longitudinal fascicle (MLF), the two locations where TAI predominantly occurs in the brainstem. Authors are confident that their selected sample of sections was representative of the tracts overall. Images were captured and digitized at a magnification of 50× (CSpT) and 25× (MLF). A 45.000 µm^2^ grid was superimposed over these regions of the CSpT and a 135,000 µm^2^ of the MLF and the length and width of the six longest axonal profiles was measured using the ImageTool v3.0 software calibrated to our light microscope and image analysis system.

### 2.6. Statistical Analysis

For each region six fields of the CSpT and of the MLF where counted in each animal and average length and width/slide was used to calculate average length and width/animal, than Student-t test was used to compare the width and length of HRP-labeled axons in both regions in vehicle treated animals and in MDL-28170 treated rats. Differences were assumed significant at a level of p ≤ 0.05.

## 3. Results

*Physiological Parameters*. In accordance with previous observations the above-described experimental protocol did not result in any significant alteration in the physiological parameters monitored during the experiment. 

*Light microscopic examination* of vehicle and drug treated animals subjected to TBI and reacted for the tracer showed well-defined reaction products within scattered axons localized in the CSpT and MLF. Length and thickness of the swollen, occasionally vacuolated, sometimes partially or totally disconnected axonal segments were measured. In the MDL-28170 treated group, both the localization and the appearance of axonal profiles were similar to that observed in the vehicle treated group; however, the length of the damaged axonal profiles appeared decreased in the CSpT in the MDL-28170 treated group, and the labeled axons in the vehicle treated group displayed a more vacuolized appearance consistent with our previous observation detecting progressive intraaxonal proteolytic alterations. The overall thickness of the traumatically injured, HRP-labeled axons appeared thicker in the MLF than in the CSpT.

**Figure 1:**
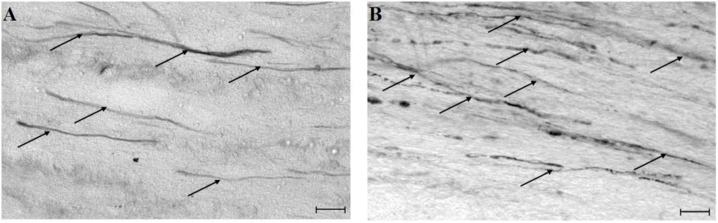
Traumatically injured axons displaying HRP-accumulation indicating axonal injury (altered axolemmal permeability) in light micrographs of the corticospinal tract **(A–B)** from injured animals. Arrows show HRP-labeled axonal profiles. Note that the length of damaged axons appears reduced in the MDL-28170-treated **(A)** compared to the vehicle-treated **(B)** section in the CSpT and vehicle treated axons display a more vacuolized appearance. (Magnification bar indicates 20 μm.

*Digital data acquisition followed by statistical analysis* confirmed that preinjury administration of MDL-28170 significantly reduced the length of the damaged HRP-labeled axonal profiles in the CSpT (from 84.2 ± 5.2 µm to 48.1 ± 3.4 µm [t *=* 2.57, df *=* 6, p < 0.04)]. There was neither significant difference in the length of labeled axons in the MLF nor in the thickness of axonal segments in either fiber tracts. Average length of damaged HRP-labeled axonal profiles in the MLF was 120.1 ± 10.1 µm in drug treated and 120.4 ± 6.2 µm in vehicle animals. In drug treated animals thickness of injured axons was 2.5 ± 0.1 µm in the CSpT and 4.9 ± 0.3 µm in the MLF, while in vehicle-treated rats it was 2.9 ± 0.2 µm in the CSpT and 5.8 ± 0.3 µm in the MLF.

**Figure 2:**
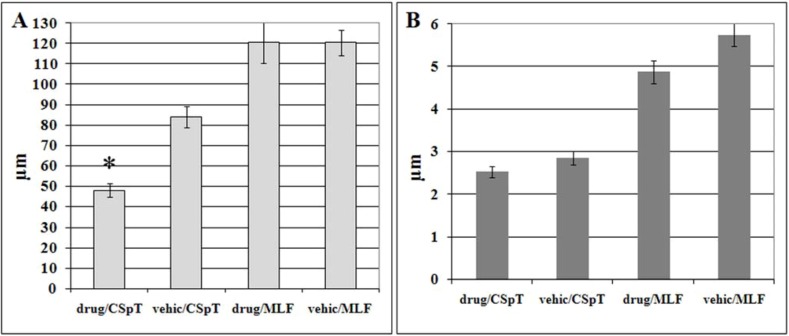
Bar chart of the mean length (**A**) and thickness (**B**) of traumatically injured axons (displaying HRP-accumulation) in the corticospinal tract (CSpT) and in the medial longitudinal fasciculus (MLF) in vehicle- (vehic) and MDL-28170-treated (drug) animals subjected to impact acceleration injury. Error bars represent standard error of mean (SEM); asterisks indicate statistically significant difference of mean density values.

## 4. Discussion

In the present study, we demonstrated that pre-injury administration of the calpain-inhibitor MDL-28170 is capable of significantly reducing TAI assessed by a tracer (HRP) targeting APA evoked by TBI. In addition to our previous observations, these results provide direct evidence for the association between CMSP and APA. Not only does this study strongly support the potential utility of calpain-inhibitors in blunting the progression of TAI, but also provides further evidence of the role of Ca^2+^-induced CMSP in TBI as previously suggested both in animals [10,17], and man [24,25].

While the present study did not utilize parallel ultrastructural analyses to define the precise subcellular targets of the MDL-28170, the use of HRP targeting specific features of TBI provide insight to its potential mode of action. As it has been alluded to previously, TBI-induced rise in intraaxonal Ca^2+^, coupled with the increase in CMSP, contributes to microtubular loss and dispersion, disrupting axonal transport kinetics, concluding in axonal transport impairment [16].

On the basis of several recent reports [5,6] a rather complex picture is developed about the pathogenesis of traumatically induced diffuse neuronal/axonal injury indicating that it is far more versatile than it has been proposed a few years ago. Nevertheless, APA, due to the shearing forces of injury, seems to be a consistent feature at least in a subpopulation of damaged axons.

The attenuation of HRP-accumulation via the use of this calpain inhibitor illustrates that these agents have a therapeutic potential in terms of inhibiting proteolytic consequences of mechanoporation-induced Ca^2+^-accumulation in TAI. The results of the current communication are novel in the context of TAI, yet, they do join a relatively large volume of literature indicating the neuroprotective properties of calpain inhibitors in various central nervous system disorders including ischemic brain injury [9,22,26] and spinal cord injury [11,27]. Our results also extend other work conducted in TBI wherein the use of calpain-inhibitors was observed to improve behavioral outcome although it did not exert neuroprotection in terms of contusional volume reduction and/or apoptotic change [28]. In a recent study we also demonstrated how pre-injury administration of the same calpain-inhibitor utilized in this work was capable of reducing the density of TAI in the rodent brainstem; now we extended and corroborated these results while also providing further data on the specific subcellular effects of calpain-inhibition and particularly on our former hypothesis on its role in APA [20].

The major limitation of the current investigation resides in the fact that the results rely on the use of a singular pre-injury injection strategy, which was mandated by the limited availability of the chosen drug. Ideally, to demonstrate potential pre-clinical relevance, it would have been of interest to observe the axonal protection afforded by calpain-inhibitor delivered in a delayed post-injury injection schedule.

While it is obvious that the inhibition of APA and other pathological events linked to Ca^2+^-induced CMSP should not be considered a panacea, further investigations to develop cell permeable and highly selective calpain inhibitors with better physical-chemical properties than those of MDL-28170 should bring us closer to the better care of TBI.

## 5. Conclusions

Increased axolemmal permeability evoked by shearing forces of TBI might play a key role in the pathogenesis of TAI. According to our observations calpain inhibitors are capable of limiting the progression and extent of axolemmal leakage most probably via inhibition of Ca^2+^-induced CMSP. Next step of our investigation should be to apply tests to correlate histological damage/improvement with outcome. While this finding requires further preclinical tests, it indicates that calpain inhibitors should be considered potential candidates in fighting TBI, the “silent epidemic”.
